# Evaluation of MCM-2 Expression in TMA Cervical Specimens

**DOI:** 10.1371/journal.pone.0032936

**Published:** 2012-04-06

**Authors:** Alcina F. Nicol, José R. Lapa e Silva, Cynthia B. Cunha, Sergio M. Amaro-Filho, Nathalia Oliveira, Beatriz Grinsztejn, Ruth Khalil, Fabio Russomano, Andrea Pires, Jonathan E. Golub, Gerard J. Nuovo

**Affiliations:** 1 Laboratory Interdisciplinary of Medical Research, Instituto Oswaldo Cruz, Rio de Janeiro, Brazil; 2 Laboratório Multidisciplinar, Clementino Fraga Filho University Hospital, Universidade Federal do Rio de Janeiro, Rio de Janeiro, Brazil; 3 Department of Infectious Disease, Evandro Chagas Clinical Research Institute, Fundação Oswaldo Cruz, Rio de Janeiro, Brazil; 4 Department of Cervical Pathology, Fernandes Figueira Institute, Fundação Oswaldo Cruz, Rio de Janeiro, Brazil; 5 Fonte Medicina Diagnostic Laboratory and Federal Fluminense University, Niterói, Rio de Janeiro, Brazil; 6 Johns Hopkins Bloomberg School of Public Health and Johns Hopkins School of Medicine, Baltimore, Maryland, United States of America; 7 Comprehensive Cancer Center, Ohio State University, Columbus, Ohio, United States of America; University of Toronto, Canada

## Abstract

**Background:**

Minichromosome maintenance proteins (MCM) are highly expressed in actively replicating cells. The need for biological markers for cervical carcinoma and its precursor lesions is emerging. Our main aim was to determine the immunohistochemical expression of MCM-2 in HIV-positive and -negative dysplastic cervical specimens.

**Methods:**

Immunohistochemical analysis of MCM-2 was performed in a total of 352 cervical TMA specimens of normal control, low-grade CIN, high-grade CIN and invasive tumor. 38 specimens were from HIV-positive women. A receiver operating characteristic (ROC) curve was constructed to determine the best cutoff to diagnose high-grade CIN and invasive cervical cancer.

**Results:**

In the progression from normal epithelium to high-grade CIN and invasive tumor we found significant differences in the MCM-2 expression (p<0.05). Based on the ROC curve of 80% with an area under the curve (AUC) of 0.78, expression of MCM-2 to diagnose high-grade CIN and invasive tumor resulted in sensitivity of 81%, specificity of 66%, a positive predictive value (PPV) of 86% and a negative predictive value (NPV) of 57%. HIV-positive cervices revealed a decreasing expression of MCM-2 in both LGCIN and HGCIN compared with HIV-negative specimens (p<0.0001).

**Conclusions:**

The present study suggests that immunohistochemical MCM-2 may not be a promising biomarker for diagnosing high-grade CIN and invasive cancer.

## Introduction

A number of potential biomarkers for cervical screening have been analyzed that appear to improve the detection of women at greatest risk for developing cervical cancer [1]. The Minichromosome maintenance proteins (MCM) have emerged as promising proliferation markers in several tumor types [2], [3]. The MCM protein family consists of 7 major isoforms (MCM2 - 8) that have similar biochemical functions and are essential for DNA replication. Dysregulated expression of MCM proteins is characteristic in a variety of proliferative and malignant conditions, leading to widespread expression [4].

Additional adjuncts are necessary to optimize the accuracy of pathologic diagnosis of cervical neoplasia, considering the interobserver and intraobserver variability for cervical dysplasia in histology. Most studies have evaluated MCM immunohistochemical expression based on the percentage of MCM staining malignant cell. However, this is a controversial issue and no standard definition for MCM expression have been developed [1]. Moreover, limited data of MCM-2 evaluation in cervical specimens are available and none in the context of HIV-1 infection. Thus we aimed to evaluate the immunohistochemical expression of MCM-2 in abnormal cervical epithelium in women with and without HIV-1 co-infection to determine if MCM-2 is a valid prognostic biomarker for cervical cancer.

## Materials and Methods

A total of 352 Tissue Micro Array (TMA) specimens of formalin-fixed, paraffin-embedded cervical tissues were immunohistochemically analyzed. Of these samples, 169 were obtained from the archive files of the Department of Pathology from the Fernandes Figueira Institute (IFF), Fiocruz, Rio de Janeiro, Brazil between March 2009 to December 2009 and included 38 cervical samples from HIV/HPV co-infected patients. The TMA blocks were constructed as previously published [5], the size of the punches were 1 to 1.5 mm diameter and there were two cores, with full thickness of the cervical epithelium. Clinical information were obtained from the record files. Another set of four TMA slides was obtained from USBIOMAX-USA (CR 804, CIN 481, BC 10021 and CR 2081), and all tested negative for HIV. Additionally, 15 single slides of low grade CIN (CIN I) were obtained from the Department of Pathology at Ohio State University – USA. The cases were chosen at random from those available in each diagnostic category. Specimens were identified by final diagnosis on the histopathology report. The study was approved by Oswaldo Cruz Foundation Institutional Ethics Review-Board.

### Immunohistochemistry analysis

Sections of 5 µm were cut on to silane-coated slides (Sigma, St. Louis, MO, USA), and processed for immunohistochemistry, as previously described [6]. We used mouse monoclonal primary antibodies against MCM2 (BD ProEx™ C, dilution 1∶400). Briefly, tissue slides were deparaffinized, and antigen retrieval was carried out by treating the sections with Target Retrieval Solution, pH 6.0 (S1699, DAKO, Copenhagen, Denmark). Primary antibody (100 µL) was applied in a humidified chamber at 4°C overnight. The LSAB system HRP (Dakocytomation, Carpinteria, CA, USA) method was adapted for immune-labeling the biotinylated link universal antibody and then the streptavidin-HRP conjugate for 30 minutes. Slides were washed three times in Tris pH 7.6 between each incubation step. Antibody binding was visualized with Fast Red Chromogen (Sigma Chemical Co.,St. Lois, MO, USA) for MCM-2. Finally, slides were counterstained with hematoxylin, dehydrated and mounted in a resinous mounting media (Merck, Darmstadt, Germany). Negative controls were done for all tissues by omitting the primary antibody.

### Immunohistochemical evaluation

The microscopic analysis of the slides was independently performed by two investigators. Digitalized photographs were taken with a Nikon COOLPIX Camera DP12, and the images stored in a computer-based software program for documentation. Quantitative results were expressed by % of stained cells/field as previously published [6]. Only cells within cervical epithelium were counted. All sections slides were assessed at 400× magnification and separately evaluated by two observers (AFN) with more than 10 years experience in immunohistochemistry and (NO). The TMA were reviewed by an experienced molecular pathologist (GN). Only nuclear staining was considered positive for MCM-2. Results were compared with the HIV serostatus of the patients.

### Semiquantification of the stained cells on the layers of cervical epithelium

Besides the quantification of total stained cells in the cervical epithelium, described above, a semiquantification analysis was done. Semiquantitative indication of the stained cells was obtained by calculating a labeling index (LI), as previously published by Freeman et al 1999 [4]. At least 200 nuclei were assessed per case. Counts were done manually, a percentage of positively stained nuclei out of the total number of nuclei were counted in representative microscopic fields. Labeling index were determined for three epithelial compartments. For each section the epithelial thirds were defined by measuring the epithelial thickness and dividing by 3: the basal and parabasal layer (1–3 layers); middle/intermediate (around 8 layers) and superficial/upper layers (5–6 layers).

### In situ hybridization and co-expression analyses

We performed HPV in situ hybridization on selected cases using a previously published protocol [6]. The HPV in situ hybridization was done for the purposes of co-expression analyses with MCM-2 in the same sections to be able to address the question as to whether productive HPV infection was driving MCM-2 expression. The co-expression analysis was done with the Nuance system as previously described [7]. in brief, the nuance system isolates the blue and red spectra of the HPV DNA and MCM-2 protein, respectively, converts it to fluorescent based signals and then “mixes” the two to determine if a given cell in making none, one, or each of the targets.

### Statistical analysis

Data analysis was carried out with SAS version 9.1 and R 2.11.1. The variables of MCM-2 expression are presented as medians and interquartile ranges. Correlation by counting cells (% stained/cells) was determined. Mann-Whitney U, Kruskal Wallis and Dunn's tests were applied to compare means of positive cells in the epithelium of all cases (controls, low grade CIN, high grade CIN and invasive cancer). The accuracy of MCM-2 expression to diagnosis high grade CIN and invasive tumor was evaluated with the use of ROC curve analyses according to standard procedures [8], [9]. A *p* value<0.05 was considered to indicate statistical significance.

## Results

### Subjects samples and histopathological data

#### HIV seropositive subjects

Cervical TMA specimens from 38 HIV-infected women were analyzed, 18 were low grade CIN; 17 were high grade CIN and 3 invasive tumors. Patients' mean age was: 36 (SD 7.4); Median (IQR) 36.5 (31–42.7).

#### HIV seronegative subjects

Cervical TMA specimens from 314 women were included in this study. Patients mean age was: 44.3 (SD 6); Median (IQR) 49 (39–50) and consisted of 54 normal controls; 24 low grade CIN (CIN I); 20 high grade CIN (CIN II/III) and 216 invasive tumors. The invasive tumors consisted of 192 Squamous Cell Carcinomas (26 histological classified as grade 1; 166 were grade 2/3); and 24 adenocarcinomas).

### Immunohistochemical data

In normal control cervices, MCM-2 expression was restricted to the basal compartment; however MCM-2 expression increased in the basal, middle and superficial layer (all compartments) in the progression from low grade to high grade CIN. The mean percentage of total MCM-2 immunopositivity cells in the cervical epithelium are depicted on Table 1.

**Table 1:** Crude table showing the % of stained cells in the cervical epithelium.

MCM-2*	Total %
**Control**	
Mean (SD)	4.7 (1.6)
Median (IQR)	0.3 (0.0–0.6)
N	54
**Low grade CIN**	
Mean (SD)	32.9 (5.2)
Median (IQR)	26.9 (0.5–68.2)
N	42
**High grade CIN**	
Mean (SD)	31.6 (5.6)
Median (IQR)	0.7 (0.6–68.1)
N	37
**Invasive tumor**	
Mean (SD)	47.6 (2.2)
Median (IQR)	58.9 (0.9–76.0)
N	219
**P-valor**	<0.0001

* Dunn's test for multiple comparisons: revealed significant differences among normal controls compared with low CIN, High grade CIN and invasive tumor; and between low CIN compared to invasive tumor.

Moreover significant differences were found among Invasive tumor and control, High grade CIN and control. Low grade CIN and control, invasive tumor and low grade CIN and between invasive tumor and high grade CIN.

**Figure 1:**
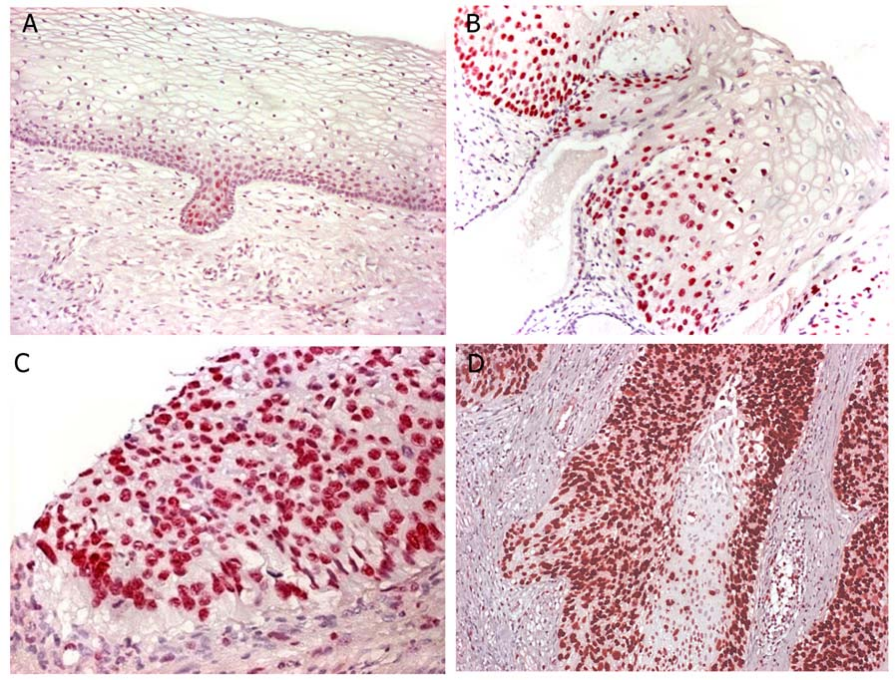
Correlation of MCM2 expression with the histological findings in the uterine cervix. Panel A shows normal cervical epithelia where MCM2 is restricted to the epithelial cells at the base of the mucosal surface. Panel B shows the MCM2 pattern in a CIN 1 lesion; note that the protein in abundantly produced by the cells towards the base, but not in the cells towards the surface that show the koilocytic change classic for CIN 1. Panel C- shows the pattern of MCM2 expression in a CIN 3 lesion. Note the strong nuclear based signal present in the full thickness of dysplastic epithelia. Panel D – shows the marked increased MCM2 expression in a invasive tumor lesion.

Interestingly, there was a marked and significant decrease in the numbers of MCM-2 positive cells in the HIV-1 positive CIN tissues compared to the HIV-1 negative CIN tissues (p<0.0001).(table 2). Dunn's test for multiple comparisons revealed differences to normal controls compared to low-grade CIN, high-grade CIN and invasive tumor. Low-grade CIN compared to high-grade CIN and invasive tumor; and between high-grade CIN and invasive tumor. All invasive tumors showed high frequency of stained cell in most neoplastic cells. The correlation of MCM-2 expression with the histological findings in CIN lesions are shown on figure 1.

**Table 2:** MCM-2 expression in HIV positive and negative cervices.

Bio-marker	MCM2		
	Manual		
	HIV- seronegative	HIV seropositive	P-valor
Control			
Media (SD)	4.7 (1.6)	-	-
Median (IQR)	0.3 (0.0–0.6)	-	
N	54	-	
Low CIN*			
Media (SD)	57.2 (4.9)	0.5 (0.5)	<0.0001
Median (IQR)	64.6 (40.8–74.6)	0.5 (0.4–0.6)	
N	24	18	
High CIN			
Media (SD)	58 (5.6)	0.6 (0.6)	<0.0001
Median (IQR)	67.2 (49.6–70.8)	0.6 (0.5–0.7)	
N	20	17	
Invasive tumor			
Media (SD)	47.5 (2.3)	54.1 (54.1)	0.7445
Median (IQR)	59.0 (0.9–76)	54.1 (52.4–55.9)	
N	216	3	

**Figure 2:**
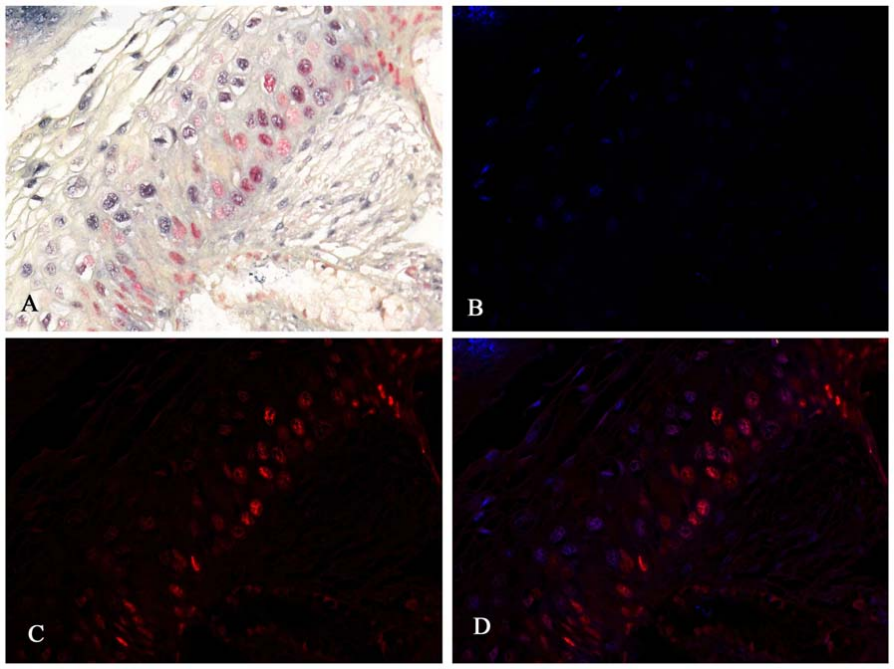
Co-expression analysis of MCM2 and HPV DNA in a CIN 1 lesion. Panel A shows the regular light microscopy view after in situ hybridization for HPV 16 DNA (blue signal) followed by immunohistochemistry for MCM 2 (red signal). The image was then analyzed by the Nuance system whereby the HPV 16 signal is fluorescent blue (panel B) and the MCM2 signal is fluorescent red (panel C). When the two latter images are overlaid (panel D) it is evident that the cells expressing MCM2 do not have detectable HPV 16 DNA and vice-versa.

Since CIN lesions are invariably associated with HPV infection, we next addressed the question as to whether the MCM-2 positive cells were primarily the cells with productive HPV infection. However, analyzing the co-labeling for HPV 16 DNA in situ and the MCM-2 by Nuance system, we found that the cells that produce MCM-2 were not the same cells where HPV 16 DNA was proliferating (figure 2). As is evident from the figure, in most cases the MCM-2 positive cells lacked the cytologic features of productive HPV infection (that is, the koilocyte), which was reflected in a very poor correlation of HPV DNA detection by in situ and co-expression of MCM-2 protein.

### **S**emi quantitative analysis of MCM 2 labeling indices

**Figure 3:**
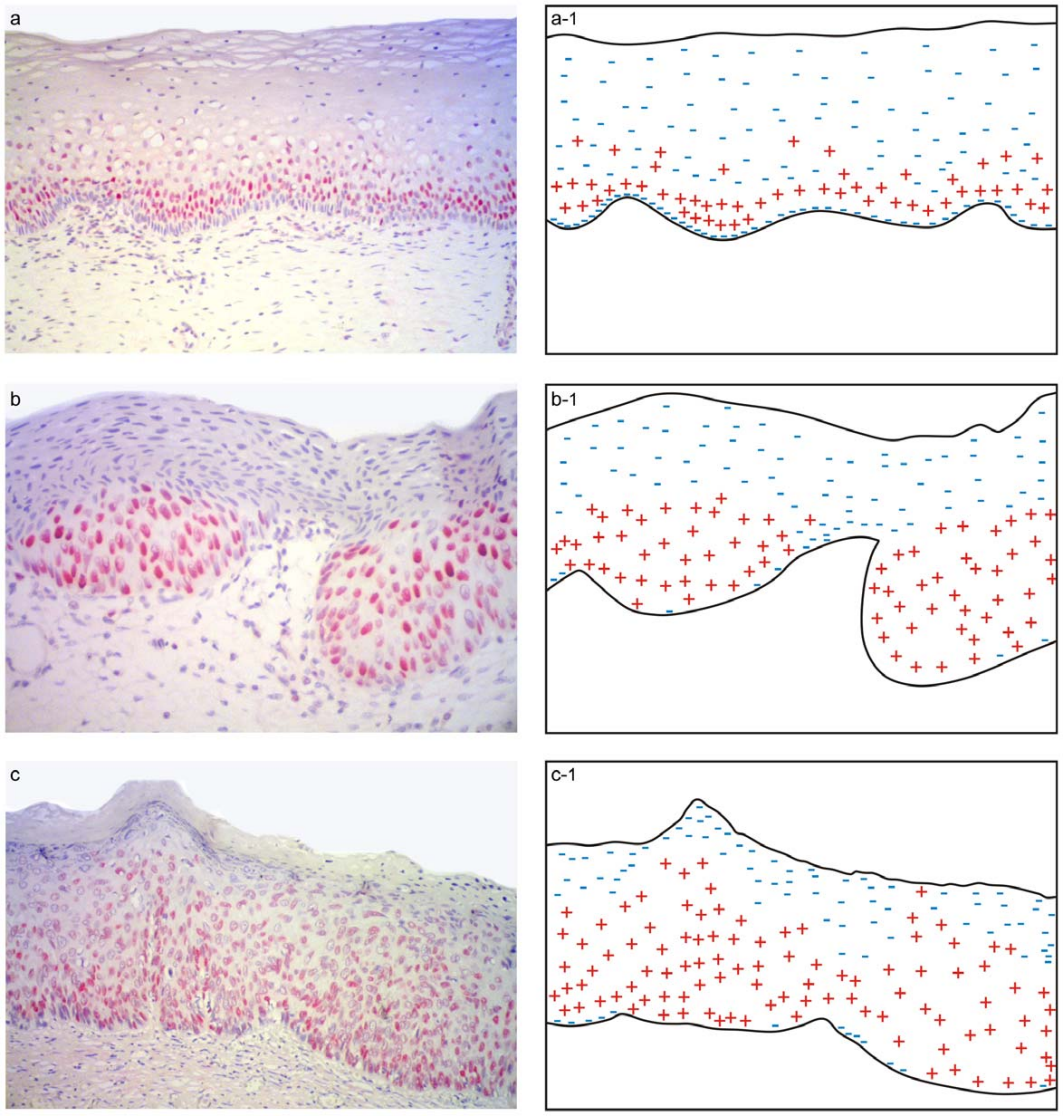
A simplified schematic representation of the different expression patterns in the three layers of the cervical epithelium. In all lesions, MCM-2 staining was present in the parabasal and intermediate layer of the epithelium. Less expression was found on the control specimens. Positive expression on the superficial layer was present in High grade CIN.

To address the question whether there is difference in MCM-2 expression in the different layers of the cervical epithelium, we did a semi quantitative counting analysis. In all lesions, MCM-2 staining was present in the parabasal and intermediate layer of the epithelium. Positive expression on the superficial layer was present in high grade CIN. On table 3 we can see the median labeling indices found (percentage of stained cells) for MCM2 in Basal/parabasal, Intermediate/middle and superficial/upper layers of control, LGCIN (CIN I) and HGCIN (CIN II/III). A simplified schematic representation of the control, LGCIN, HGCIN expression patterns is depicted in Figure 3.

**Table 3:** Semiquantitative analysis of MCM 2 labeling indices (LI) and range of immunopositivity.

	Basal/Parabasal layer % (range)	Intermediate layer % (range)	Superficial layer % (range)
**Control**	>40 (40–60)	>50 (50–60)	0
**LG-CIN HIV-neg**	>80 (80–87)	>40 (42–65)	0
**LG-CIN HIV-pos**	>50 (50–83)	>25 (25–50)	0
**HG-CIN HIV-pos**	>80 (80–89)	>85 (85–95)	>30 (30–56)
**HG-CIN HIV neg**	>90 (98–99)	>90 (99–100)	>25 (0–50) *

* 2/20 HG-CIN specimens had no (0) immunopositivity cells.

### Evaluation of sensitivity and specificity of MCM-2 in diagnosing high-grade CIN

**Figure 4:**
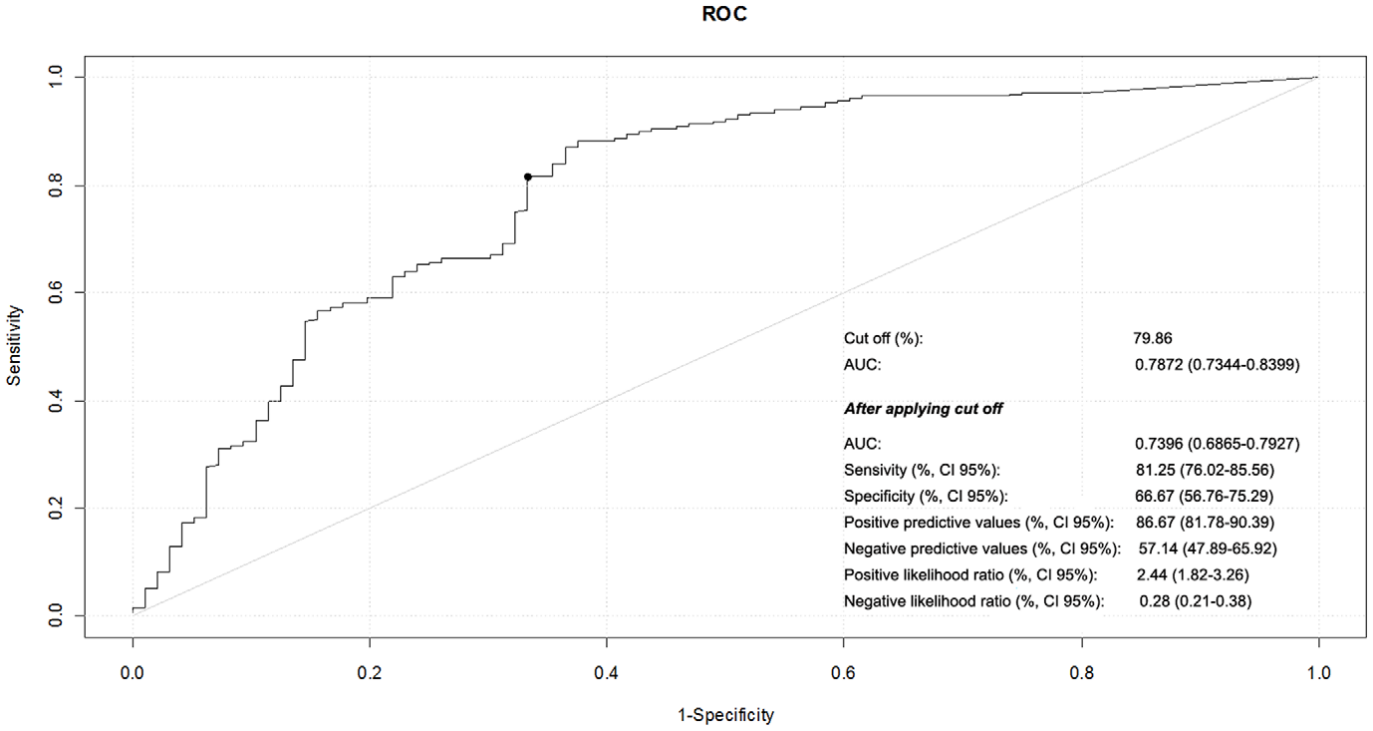
Receiver operating characteristic curve (ROC) for the overall performance of MCM-2 to predict high-grade CIN and invasive tumor.

Based on the receiver operating characteristic (ROC) curve of 80% with an area under the curve (AUC) of 0.78, expression of MCM-2 to diagnose high-grade CIN and invasive tumor in the HIV-1 negative CIN cases resulted in higher sensitivity (81%), however moderate specificity (66%), positive predictive value (PPV), (86%) and negative predictive value (NPV), (57%). The results of ROC curve, sensitivity and specificity calculations are depicted in Figure 4.

## Discussion

In the present study we evaluated MCM-2 in normal and abnormal cervical biopsies; we found an increasing percentage of positive cells following the severity of CIN (p<0.0001), in accordance with previous report [10]. MCM immunocytochemistry has great promise as a technique for cervical screening in developing countries [10]. The search for biological markers for predicting cervical carcinoma and its precursor lesions is a current priority. Biomarkers that can accurately predict the future development of disease are powerful screening tools that can potentially lead to earlier detection and better prognosis for patients with cervical precancerous lesions. MCMs are useful markers of cell-cycle entry and are abundant in the nucleus throughout the cell cycle and lost on cycle exit with rapid loss in differentiating cells. Moreover, MCM-2 has been studied in a wide range of human malignancies and has been associated with tumor histopathological grade in several malignancies, including colon, oral cavity, ovarian and urothelial carcinoma [1]. Significant associations with MCM-5 and MCM-7 with histopathological grade have been observed in cervical, ovarian and urothelial tumors, suggesting that each member of MCM protein have specific functions [1]. The majority of studies so far are restricted to MCM-2, denoting that future research should be extended to the other members of the MCM family. We found that MCM-2 stained cells were confined to the basal proliferate layer in the normal cervix. However in the progression to CIN, higher expression of this marker was found in all epithelial compartments, paralleling the entry of cytological abnormal cells in the entire width of the epithelium in high grade CIN with the exception of the cells that showed koilocytic changes and, as expected, high copy HPV DNA. This suggests that productive HPV infection which is typical especially of CIN 1 and CIN 2 lesions is associated with the down-regulation of MCM-2 expression. The finding that MCM-2 positive cells are found above the basal zone in CIN lesions is in agreement with one previous study [4].

To our knowledge no studies are available about MCM-2 expression in HIV abnormal cervices and few studies have reported the use of these biomarkers in the pathology of HIV positive anal dysplasia [11], [12]. In our previous work [13] we demonstrated significant altered expression of regulatory and cell cycle proteins in the cervix from HIV-infected women, compared to HIV non-infected cervices. Surprisingly, in this study we found a marked decrease in the MCM-2 expression in the HIV positive cervices in the CIN 1 and CIN2/3 lesions compared to HIV negative, however the MCM-2 expression markedly increased in the invasive tumor from women with HIV-confection. The basis for this observation will require more study. It is established that HIV-1 per se rarely infects the CIN cells [14]. However, as we and others have shown, co-infection by HPV and HIV-1 results in marked cytokine profile changes in the lamina propria of the cervix which could translate into decreased MCM-2 expression in the overlying CIN cells.

Interestingly, our co-labeling data revealed that MCM-2 stained cells are mutually exclusive in a given lesion from cells which there is detectable HPV16 DNA proliferation. This suggests that productive HPV infection, as characterized by high copy HPV DNA visualized by in situ hybridization, short-circuits the high proliferation index of the cells migrating from the basal zone. One may speculate that this may be advantageous to the spread of the infectious HPV as it allows more viral DNA synthesis at the expense of host cell DNA synthesis.

Most studies have evaluated MCM immunohistochemical expression based on the percentage of MCM staining malignant cells. However, this is a controversial issue and reports from prior studies are quite variable [4], [10], [11]. There is a need for a standard definition for MCM expression and which method provides the best cut off points for considering a staining pattern as positive [1]. In this context it is necessary to define a standard criterion for MCM expression in order to consider MCM as a diagnostic and prognostic factor in routine clinico-pathological settings

In the present study, the accuracy of MCM-2 expression to diagnosis high grade CIN and invasive tumors was evaluated with the use of ROC curves. ROC analysis addresses the variance of sensitivity and specificity and the AUC is the most commonly used index of performance associated with ROC analysis [8]. According to standard procedures to achieve optimal sensitivity and specificity and the cutoff of 80%, an AUC of 0.78 was established. Our overall data resulted in relatively high sensitivity (81%) and moderate specificity (66%), PPV (86%) and NPV (57%), to diagnose high-grade CIN and invasive tumors. The AUC can be interpreted as the average sensitivity over the entire range of possible sensitivities [9]. One previous report found that increasing the positive cutpoint for one specific marker increased its specificity and accuracy for HGCIN [15].

There are several scales for AUC value interpretation, but in general, ROC curves with an AUC ≤0.75 are not clinically useful [8], [9]. Therefore the AUC of 0.78 found on the present study suggests that MCM-2 is a borderline biomarker in diagnosing high grade cervical dysplasia and invasive tumors. In the present study we performed two different counting cells analysis. Our semiquantification analysis of MCM-2 labeling index in the three epithelium layers, did not reveal immunoreactive stained cells for control as well for LGCIN in the superficial layer, however on HGCIN, positive stained cells was seen (range 0–56), in both HIV positive and negative cervices suggesting that MCM-2 probably may have a role to distinguish LG-CIN from HG-CIN. However more CIN specimens should be evaluated to confirm MCM2 as a useful adjunct tool in distinguishing LG-CIN from HGCIN. The crude total epithelium counting cell analysis found that our study differ from others [10], [11], [16] that demonstrated MCM as a useful biomarker for dysplasia and malignancy.

However those previous reports only presented sensitivity and specificity of MCM-2. Neither PPV or NPV were provided, nor any ROC curves to establish the best cut point. Furthermore it is important to note that ROC performance may change when the diagnostic test is applied in different clinical situations or under different phases of test development [8]. In fact it will be rare for a diagnostic test to have both 100% specificity and sensitivity. One recent review concluded that there is no one biomarker which can predict the potential to progress of a particular cervical lesion, emphasizing that a combination of markers is more useful than a single marker [17]. A limitation of our study was the relatively small number of HIV positive samples, limiting subgroup analyses and resulting in wide confidence estimates. Given this limitation, our results should be viewed as hypothesis generating. Further research is warranted to evaluate the potential role of MCM-2 as diagnostic biomarker for CIN. In sum, our data suggests that MCM2 may not be a useful indicator of CIN in general or invasive cervical cancer because: 1) it has a relatively low specificity (66%) plus NPV (57%) and 2) the expression of MCM2 can be markedly influenced by non-HPV related factors, such as co-existent HIV-1 infection as shown in this.
