# Correction: Evaluation of MCM-2 Expression in TMA Cervical Specimens

**DOI:** 10.1371/annotation/1c0339c8-6f44-4e63-b3a6-bf49b18a0292

**Published:** 2012-04-13

**Authors:** Alcina F. Nicol, José R. Lapa e Silva, Cynthia B. Cunha, Sergio M. Amaro-Filho, Nathalia Oliveira, Beatriz Grinsztejn, Ruth K. Friedman, Fabio Russomano, Andrea Pires, Jonathan E. Golub, Gerard J. Nuovo

There was an error in the seventh author's name. The author's correct name is Ruth Khalili Friedman. The author's contributions were: Analyzed the data: RKF. 

